# Stabilisation of Collagen Sponges by Glutaraldehyde Vapour Crosslinking

**DOI:** 10.1155/2017/8947823

**Published:** 2017-05-09

**Authors:** Yong Y. Peng, Veronica Glattauer, John A. M. Ramshaw

**Affiliations:** CSIRO Manufacturing, Bayview Avenue, Clayton, VIC 3169, Australia

## Abstract

Glutaraldehyde is a well-recognised reagent for crosslinking and stabilising collagens and other protein-based materials, including gelatine. In some cases, however, the use of solutions can disrupt the structure of the material, for example, by causing rapid dispersion or distortions from surface interactions. An alternative approach that has been explored in a number of individual cases is the use of glutaraldehyde vapour. In this study, the effectiveness of a range of different glutaraldehyde concentrations in the reservoir providing vapour, from 5% to 25% (w/v), has been explored at incubation times from 5 h to 48 h at room temperature. These data show the effectiveness of the glutaraldehyde vapour approach for crosslinking collagen and show that materials with defined, intermediate stability could be obtained, for example, to control resorption rates in vivo.

## 1. Introduction

Glutaraldehyde (GA) has been used extensively as a crosslinking agent for collagen-based biomedical materials [1]. This includes its use in tissue based devices such as heart valve replacements [2, 3] and for tissue biosynthetic products [4]. Also, it has been used for products based on purified collagen, including collagen pastes [5] and freeze dried collagen sponges [6]. Most recently it has been examined for stabilisation of recombinant collagen products [7].

Despite its extensive use in medical products, concerns remain still about its potential cytotoxicity [8–10] and it being a causative agent of nonspecific tissue calcification [11]. Certainly, nonspecific calcification of biologically derived heart valves is a significant issue and leads to loss of function and the need for revision [12], although catastrophic failure is not a normal issue. Various methods have been examined to reduce this calcification [13, 14]. It has been suggested that the cytotoxicity and calcification arise from the propensity of GA to form reactive polymers, particularly at the neutral pH conditions normally used for tissue and collagen stabilisation. At acidic conditions, for example, around pH 3-pH 4, GA is found predominantly as a monomer but taking the reagent to neutral pH leads to formation of polymeric forms [15]. Crosslinking will occur at pH 4, but it is slow and gives materials of lower thermal stability [16].

Other approaches have looked at ways to minimise the amount of GA polymer present during neutral pH stabilisation. One approach is to stabilise collagen with GA at an elevated temperature, for example, up to 50°C [17], which is less than typical tissue shrinkage temperatures. Examination of GA solutions at elevated temperature, for example, by NMR spectroscopy, shows an increase in free aldehyde content [18].

Another approach is to use GA vapour, which has been used in the preparation of collagen-based biomedical materials [19–21]. In particular, it has been used for samples that are initially hard to handle without damage, including sponges from bacterial collagens which may disperse in solvents [7]. More studies, however, have been done on the denatured form of collagen, gelatine, especially on electrospun gelatine materials [22–24], as well as on a number of protein composite materials that include notionally collagen or gelatine [25, 26]. Electrospun gelatine can also be formed unintentionally, from electrospinning of collagen in harsh solvents [27], although more recently benign solvents have been used for electrospinning of collagen without associated denaturation [28, 29]. Electrospun films of collagen or gelatine are frequently quite fragile, so the use of vapour phase crosslinking has clear advantages over solution methods.

Previous GA vapour stabilisation studies have used a wide variety of conditions with variations in time of exposure, GA concentration in the reservoir, and reaction temperature, with no preferred procedure emerging. In the present study, we have examined a range of conditions, all at room temperature to understand better the extent and rate of crosslinking that can occur.

## 2. Materials and Methods 

### 2.1. Collagen Sponge Preparation

Bovine type I collagen was purified from yearling hides obtained from a local abattoir using the well-established method [30] of pepsin solubilisation of minced, unhaired dermis, using 1 mg/ml pepsin (Sigma-Aldrich) in 100 mM acetic acid (Merck). Fractionation of different collagens and purification of type I collagen was by NaCl precipitation in acetic acid and then at pH 7.2, as previously described [30, 31]. Purified bovine type I collagen at 10 mg/ml in 50 mM acetic acid was used to prepare freeze dried collagen sponges, around 3 mm thick. Circular samples 6 mm in diameter for glutaraldehyde (GA) treatment and analysis were cut from the sponge sheets with a biopsy punch. Except where otherwise noted all other chemicals were of the highest grade readily available and obtained from Merck (Victoria).

### 2.2. Glutaraldehyde Treatments

For GA vapour crosslinking, GA was obtained as a 50% (w/v) stock solution (ProSciTech, Thuringowa, QLD). For crosslinking, >20 ml GA solutions of various concentrations made by diluting the stock GA solution with water, from 5% up to 25% (w/v), were held in the lower part of glass desiccators in 70 mm dishes. Cut sponge disks were held in glass dishes above these solutions, allowing ready access to vapour, and the desiccator lid was placed to seal the chamber. Samples were removed at selected times, up to 48 h, of GA vapour exposure. Control samples, with no GA treatment, were handled in a similar manner but were held over water and had no exposure to GA. Samples for Differential Scanning Calorimetry (DSC) analysis were washed with 40 mM glycine for 3 h and then washed in MilliQ water and air dried. Other samples were held isolated, open to a stream of clean air for at least 12 h prior to any further analysis.

### 2.3. Differential Scanning Calorimetry

The thermal stability of untreated and GA vapour treated collagen disks were determined by DSC using a Mettler Toledo DSC821 instrument. The collagen sponge disks were between 0.8 and 0.9 mg each and were rehydrated in phosphate buffered saline (PBS) prior to analysis. A heating rate of 5°C/min was used. Data were averaged from separate sample determinations, with at least 2 determinations for each condition. The range of values obtained was typically around 1°C for each condition tested.

### 2.4. Scanning Electron Microscopy

Samples were examined after Ir coating (30 sec, 60 mA) using a Cressington 208HR sputter coater using a Zeiss Merlin Gemini 2 FESEM instrument.

## 3. Results and Discussion

GA is a widely used crosslinking agent for collagen, gelatine, and many other proteins that is normally used at a dilute concentration, for example, 0.1 to 2.0% (w/v), in aqueous solution [1] or less frequently in organic solvents [32]. In some cases, the nature of the sample makes it unsuitable for stabilisation in solution. In these instances, using GA vapour has proved a suitable alternative.

Previously a wide range of isolated treatment conditions have been used (Table 1), in which variations in temperature and in the GA concentration in the vapour reservoir have been used. The present study has compared the effects of concentration and time variations on the effectiveness of collagen crosslinking. The effectiveness of the crosslinking has been determined by the increase in the collagen melting temperature (*T*_m_, denaturation temperature). This method allows moderately rapid, reproducible determinations, but the high rate of heating, 5°C/min, can lead to a slight increase in values, especially at lower temperatures, compared with methods that use a lower heating rate, where a *T*_m_ around 4°C lower may be observed.

**Figure 1:**
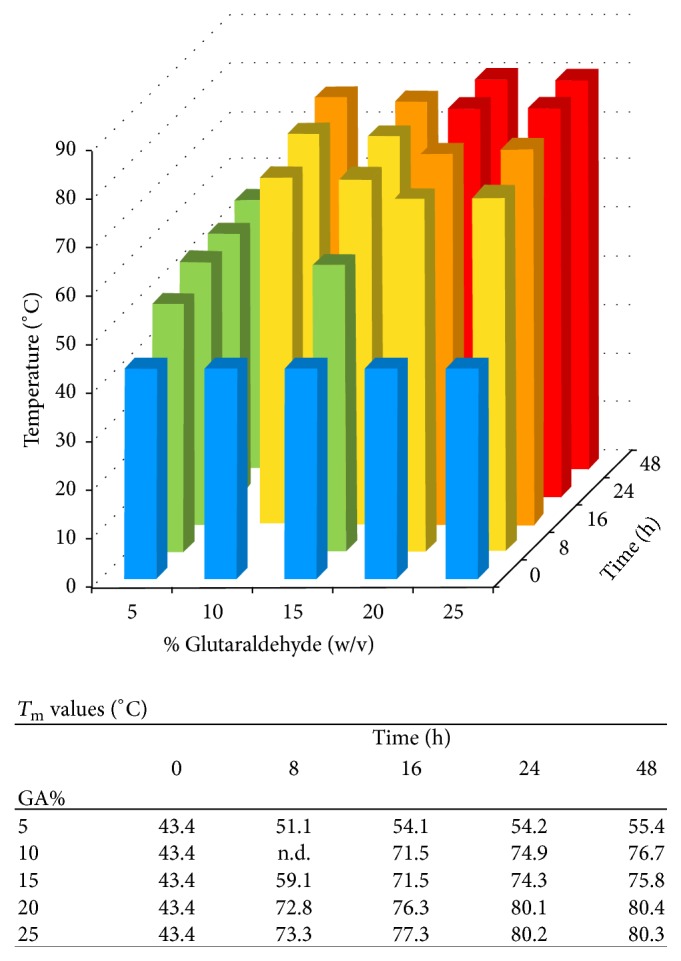
Bar diagram, showing the melting temperature, *T*_m_, of collagen sponges treated with varying amounts of aqueous GA vapour for various time periods. Red bars indicate *T*_m_ > 80°C, orange bars indicate 75°C < *T*_m_ < 80°C, yellow bars indicate 70°C < *T*_m_ < 75°C, green bars indicate 50°C < *T*_m_ < 70°C, and blue bars indicate *T*_m_ < 50°C, as found for the average value of control untreated sponges. A single average *T*_m_ value for control untreated samples was obtained and this average value was used in each of the different % GA treatment series. *T*_m_ values are given below the diagram. n.d.: not determined.

The present study has examined concentrations ranging from 5% (w/v) GA up to 25% GA (w/v) in aqueous solution and incubation time up to 48 h (Figure 1). The temperature used was room temperature, which had most frequently been used by others (Table 1). Previously, higher temperatures have been used in some studies [20, 22], where increased speed of crosslinking is expected, in part from the increase in GA vapour pressure. For example, the GA vapour pressure increases around 7-fold for a 15% (w/v) solution between room temperature (20°C, 32 ppm) and 40°C (226 ppm) [42].

These present data show that essentially full crosslinking, *T*_m_ > 80°C, can be achieved by using 20% or 25% (w/v) reservoir solutions for 24 h or 48 h. This is consistent with previously report *T*_m_ data [20, 21], where a *T*_m_ of >80°C was reported for a collagen sponge over 25% (w/v) GA at room temperature for 24 h [21] and similarly a *T*_m_ of >80°C was reported for a collagen film over 8% (w/v) GA at 37°C after 8 h [20]. *T*_m_ provides a good quantitative measure for crosslinking but is not always reported. Often, the physical appearance and stability of materials are quoted under varying conditions, such as in acid solution. For gelatine samples, a *T*_m_ cannot be given as the gelatine is already denatured (from collagen). The efficiency of crosslinking for GA treated gelatine samples can be estimated by examining the stability of the material to proteolysis. However, the conditions used often vary between studies, making comparisons difficult.

At lower reservoir concentrations of GA, full crosslinking did not seem to occur, even after 48 h incubation time (Figure 1). Further, it appeared that for lower concentrations the extent of crosslinking, as shown by *T*_m_ values, was appearing to approach a maximum value dependent on the concentration being used (Figure 1). Samples incubated over 5% (w/v) GA showed *T*_m_ values in the mid-50°C range after 24 h, and these values did not increase much after 48 h incubation. Samples over 10% and 15% (w/v) GA solutions may also be approaching maximum values which are in the mid-70°C range (Figure 1) and lower than the *T*_m_ values of >80°C found with higher GA concentrations. These apparent plateau values dependent on the concentration of GA in the reservoir have the potential to provide sample series of varying crosslinking, for example, for studies on resorption rate, similar to those obtained from the use of different GA concentrations in solution stabilisation [43].

**Figure 2:**
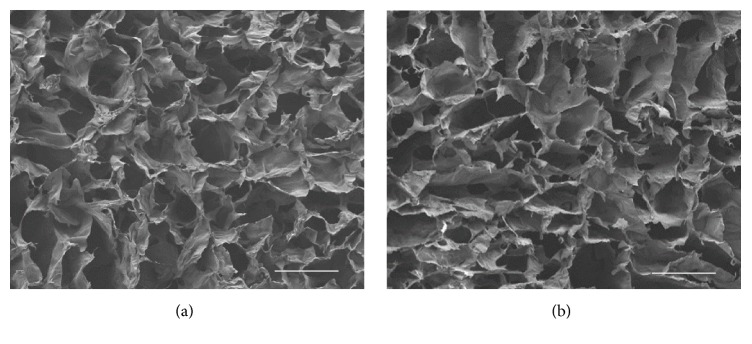
FESEM examination of collagen sponges. (a) Control untreated sponges, (b) GA vapour stabilised sponge; 25% (w/v) GA, 24 h at room temperature. Bar = 100 *μ*m.

GA vapour crosslinking has the advantage for any porous sample that by avoiding surface tension and repeated freeze drying that are found with solution approaches the use of vapour leads to negligible changes to the collagen organisation and topology. SEM studies (Figure 2), show little if any changes in the collagen sponge structure in control untreated material (Figure 2(a)) and one extensively stabilised (20% (w/v) GA, 24 h) by GA vapour (Figure 2(b)).

In the present study, we have examined purified collagen with no additions. GA vapour stabilisation can also be used for composite materials based on collagen and gelatine. These include, for example, mixtures of collagen or gelatine with other proteins [25, 26], or with other components including carbohydrates [44], polymers [45], and mineral [46].

In addition to mixtures, collagen and gelatine can be used as external coatings during coaxial spinning [47].

Several previous studies have used GA vapour crosslinking collagen-based materials for cell growth, and these studies have consistently shown that the resultant crosslinking is not cytotoxic [7, 21, 33–35, 37], even when higher GA concentrations are used at longer time points [7, 21, 33, 35]. This is consistent with the GA being reactive as the monomer and not allowing a significant build-up of polymers in the stabilised material. Other studies have shown the enhanced mechanical properties arising from GA vapour crosslinking fabricated of materials [19, 35, 36].

## 4. Conclusion

The present study has demonstrated the effectiveness of GA crosslinking over a range of conditions. It has shown that essentially full crosslinking can be obtained for collagen sponges with treatment with 20% or 25% GA vapour for 24 or 48 h at room temperature. Intermediate degrees of crosslinking may be obtained by varying the GA concentration. These observations, and the understanding of the variation in crosslinking through changes in GA concentration and time, should be useful in designing the preferred crosslinking characteristics for collagens and gelatines and in composites based on these protein materials. The use of GA vapour crosslinking is particularly useful for porous materials that are not easily handled, providing stability. Subsequently, additional solution based crosslinking could be used to augment the stability if necessary or to introduce chemical modifications while maintaining a stable structure.

## Figures and Tables

**Table 1:** Examples of previously reported conditions for GA vapour stabilisation of collagen and gelatine materials.

Substrate	Format	Temperature	% GA	Time	Reference
Collagen (Limed bovine)	Reconstituted Fibrils	RT	25%	24 h, 48 h	Kato et al. [19]
Collagen (Bovine)	Reconstituted Fibrils	RT	25%	96 h	Law et al. [33]
Collagen (Bovine)	Film	37°C	8%	Various 3 h to 72 h	Barbani et al. [20]
Collagen (Bovine)	Electrospun mat	RT	(Not stated)	24 h	Matthews et al. [34]
Collagen (Bovine)	Sponges	RT	25%	4 h, 8 h, 24 h	Lickorish et al. [21]
Collagen (Bovine)	Reconstituted Fibrils	RT	25%	Various, 1 h to 24 h	Rho et al. [35]
Collagen (Bovine)	Electrospun mat	RT	25%	24 h	Yang et al. [36]
Bacterial Collagen	Sponge	RT	20%	18 h	Peng et al. [7]
Collagen (Bovine)	Electrospun mat	RT	25%	8 h	Takeda et al. [37]
Gelatine (Fish skin)	Electrospun mat	37°C	50 vol%	3 h	Songchotikunpan et al. [22]
Gelatine	Electrospun mat		0.5%	19 h	Sisson et al. [38]
Gelatine (Porcine)	Electrospun mat	37°C	“Saturated”	5 min	Dheraprasart et al. [39]
Gelatine (A-type, B-type)	DHT-treated Electrospun mat	4°C	0.06% in acetone/HCl	48 h	Ratanavaraporn et al. [40]
Gelatine (A-type)	Electrospun mat	RT	1.5% in EtOH	48 h	Zha et al. [23]
Gelatine (Fish skin)	Electrospun mat	40°C	5%	Various, 0.5 h to 24 h	Gomes et al. [24]
Gelatine (Fish skin)	Electrospun mat	40°C	5%	5 h	Gomes et al. [41]

RT: Room temperature.
